# Process evaluation for complex interventions in primary care: understanding trials using the normalization process model

**DOI:** 10.1186/1471-2296-8-42

**Published:** 2007-07-24

**Authors:** Carl R May, Frances S Mair, Christopher F Dowrick, Tracy L Finch

**Affiliations:** 1 Institute of Health and Society, Newcastle University, 21 Claremont Place, Newcastle upon Tyne, NE2 4AA, UK; 2 Division of General Practice and Primary Care, University of Glasgow, Glasgow, UK; 3 School of Population, Community and Behavioural Sciences, University of Liverpool, Liverpool UK

## Abstract

**Background:**

The Normalization Process Model is a conceptual tool intended to assist in understanding the factors that affect implementation processes in clinical trials and other evaluations of complex interventions. It focuses on the ways that the implementation of complex interventions is shaped by problems of workability and integration.

**Method:**

In this paper the model is applied to two different complex trials: (i) the delivery of problem solving therapies for psychosocial distress, and (ii) the delivery of nurse-led clinics for heart failure treatment in primary care.

**Results:**

Application of the model shows how process evaluations need to focus on more than the immediate contexts in which trial outcomes are generated. Problems relating to intervention workability and integration also need to be understood. The model may be used effectively to explain the implementation process in trials of complex interventions.

**Conclusion:**

The model invites evaluators to attend *equally *to considering how a complex intervention interacts with existing patterns of service organization, professional practice, and professional-patient interaction. The justification for this may be found in the abundance of reports of clinical effectiveness for interventions that have little hope of being implemented in real healthcare settings.

## Background

Getting new ways of delivering and organizing healthcare into practice is a problem. In recent years governments and healthcare providers across the advanced economies have been concerned with improving the technological and organizational capabilities of health services. At the same time, they have sought to measure the outcomes and costs of these interventions, in the face of constant and increasing fiscal pressures and their political consequences [1]. One result of these political and economic pressures has been the emergence of new fields of health research, especially health technology assessment and health services research, that bring together clinical, social science and statistical researchers and ally them with policy interests that demand the clinical and economic evaluation of already discovered treatments, technologies and professional or organizational interventions [2,3] and which emphasize the importance of outcomes.

But as outcomes research has become more important, its practitioners have also increasingly faced the problem of conducting evaluation studies – often using randomized controlled clinical trials – of *complex *interventions. In 2000, the UK Medical Research Council (MRC) published its *Framework for the Development and Evaluation of Complex Interventions *[4]. This provides an internationally accepted definition of complex interventions, and a robust guide to the design of such trials.

Complex interventions in health care, whether therapeutic or preventative, comprise a number of separate elements which seem essential to the proper functioning of the interventions although the 'active ingredient' of the intervention that is effective is difficult to specify. (...) Complex interventions are built up from a number of components, which may act both independently and interdependently. The components usually include behaviors, parameters of behaviors (e.g. frequency, timing), and methods of organizing and delivering those behaviors (e.g. type(s) of practitioner, setting and location) [4].

The MRC framework emphasizes a staged approach to establishing the feasibility of interventions before embarking on large-scale evaluations. But it also recognizes that the organizational and technical *processes *of implementing and delivering a complex intervention require attention and understanding, and that these should also be a focus of evaluation. The question of process evaluation draws attention not only to questions of measuring effectiveness, but also to problems of understanding the workability and integration of interventions in settings that are themselves dynamic and complex [5,6].

The development of process evaluations is important, but is not simply a matter of developing the range of research techniques by which they might be accomplished. The development of conceptual models that provide interpretive frameworks for process evaluations is also important. This paper supports such a move by outlining and applying a new applied theoretical model for understanding and evaluating the implementation of complex interventions. The Normalization Process Model (NPM [7]), is an evaluation model that asks what people *do *to make a complex intervention workable, and to integrate it in practice. The paper develops this by first discussing the development of the theoretical model, and then applies it to two case studies of complex trials that combine both treatment and organizational interventions in primary care. In the conclusion, the implications of such models for the development of process evaluations are discussed.

## Method

The MRC framework provides a methodological rather than an explanatory approach to evaluating complex interventions. Studies that have employed the framework, like those conducted by Blackwood [8], or Robinson [9], have therefore tended to adopt a procedural rather than a theoretical approach to the problem of intervention complexity. Similar methodological or procedural approaches are encountered with other frameworks that explore, for example, empirically identified facilitators and barriers to the successful implementation of complex interventions [10-12]. This means that we have to look elsewhere for conceptual models that might help us to understand and *predict *problems of workability and integration. In broad terms, these fall into two kinds: (a) psychological models that focus on individuals' intentions and motivations as the prime movers in implementation processes [13-15]; and (b) sociological models that focus on collective action and relational behavior [16-19].

Sociological perspectives offer vital insights into how humans interact, understand, perform and organize work *collectively *in healthcare. A conceptual model that drew these insights into the field of health services research and health technology assessment is a valuable contribution to the development of process analyses because it focuses on collaborative *work *rather than individual *motivation*, and on *co-operation *rather than *intention*. Of course, psychological models are designed and refined within frameworks of prospective experimental testing and statistical analysis, and therefore seem well suited to application in complex trials without extensive conceptual or methodological modification [20]. Sociological theories are developed, in contrast, largely by interpretive means [21], and so they need to be translated into terms that have a better fit with the specific demands of process evaluations, and the requirement for prospective or predictive tests that stem from these. In this context, the NPM is a sociological model that is suitable for *prospective *process evaluations of complex interventions.

The model proposes that while measurable effectiveness or superiority in outcomes has obvious importance, the success of a complex intervention must also be understood in relation to the *workability *and *integration *of it components in practice. This stems from the longstanding observation that the performance of everyday tasks is made possible by their routinization over time, and their assimilation or embedding as taken-for-granted elements of everyday action [22-24]. Thus, a complex intervention that is completely workable and integrated becomes routinely embedded in health care work, and minimally disrupts social relations and behavior around it. The work of normalization is thus about achieving *ecological success*, and not clinical or cost effectiveness.

The development of the model, and a detailed account of its constructs, their dimensions and components, has been given elsewhere [7]. In its simplest form, it proposes that complex interventions are implemented in processes in which the collective action and interactions of patients, professionals and others are governed by four factors. Each factor suggests a proposition that may be applied to the assessment and evaluation of an intervention

### (i) Interactional workability

This refers to how work is enacted by the people doing it. A complex intervention will affect co-operative interaction over work (congruence), and the normal pattern of outcomes of this work (disposal). Therefore:*a complex intervention is disposed to normalization if it confers an interactional advantage in flexibly accomplishing congruence and disposal of work*.

### (ii) Relational integration

This refers to how work is understood within the networks of people around it. A complex intervention will affect not only the knowledge required by its users (accountability), but also the ways that they understand the actions of people around them (confidence). Therefore:*a complex intervention is disposed to normalization if it equals or improves accountability and confidence within networks*.

### (iii) Skill-set workability

This refers to the place of work in a division of labor. A complex intervention will affect the ways that work is defined and distributed (allocation), and the ways in which it is undertaken and evaluated (performance). Therefore: *a complex intervention is disposed to normalization if is calibrated to an agreed skill-set at a recognizable location in the division of labor*.

### (iv) Contextual integration

This refers to the organizational sponsorship and control of work. A complex intervention will affect the mechanisms that link work to existing structures and procedures (execution), and for allocating and organizing resources for them (realization). Therefore: *a complex intervention is disposed to normalization if it confers an advantage on an organization in flexibly executing and realizing work*.

Set out in this way, the model offers a set of general descriptions of those factors that might affect normalization processes and outcomes in the context of a trial or other evaluation of a complex intervention, and that might also be expected to affect non-experimental management interventions in healthcare settings. It also offers a set of propositions that can form the basis of testable hypotheses about observable activities with measurable outcomes. In the NPM, it is collective action and not individual motivation that is the focus of evaluating complex interventions and assessing their probable outcomes. The next section of the paper explores its application to two bodies of research around the effectiveness and implementation of complex interventions – problem solving therapies for people with depression and nurse-led heart failure clinics in primary care.

## Results

So far, we have defined normalization and the factors that govern it. Because the NPM is about what different groups of people collectively do in healthcare settings rather than what they individually intend to do, it proposes that most healthcare work – no matter how autonomous the individual practitioner – is undertaken in the context of complex and dynamic *collective *interactions. Case studies of randomized controlled trials of complex interventions in primary care form useful opportunities to explore at a *general *level how the NPM might be applied. In what follows we draw on two such examples. These are:

**a. **Trials of Problem Solving Therapies (PST) for psychosocial problems. This is a new treatment modality that also involves changes in the organization and division of labor in healthcare;

**b. **A trial of a nurse-led clinic for the management of Chronic Heart Failure that involved changes in the division of labor (shifting work from primary care physicians to nurses) and the structure of work itself, in the implementation of a clinical guideline.

These examples provide a focus for considering two of the major *types *of chronic health problems that health care systems respond to – chronic psychosocial problems, and chronic and degenerative organic disease.

### Evidence for primary care interventions

In both of the cases under examination there is strong evidence from different kinds of *outcomes *studies that community based interventions are clinically effective. The least contentious of these is Chronic Heart Failure. This is a common condition, affecting 0.8 – 3.9 % [25,26] of the general population and 8–10 % of those over 65 years of age. It represents a major public health problem because of its associated high levels of morbidity and mortality and negative impacts on quality of life. Moreover, it is a malignant condition with a poor prognosis and life expectancy worse than most common cancers [27]. Over recent years there have been many important advances in the approach to both diagnosis and management of heart failure, particularly heart failure due to left ventricular systolic dysfunction. The importance of accurate diagnosis has been emphasized and a number of therapeutic agents, particularly angiotensin converting enzyme inhibitors, beta blockers and spironolactone have been shown in large scale randomized controlled clinical trials to improve morbidity, mortality and quality of life [28,29]. These trials have led to well founded clinical guidelines [30-32] which make clear recommendations for both diagnosis and treatment of people with this condition. However, despite the evidence and the widespread availability and dissemination of these clinical guidelines the management of heart failure remains suboptimal [33]. There are a range of explanations for this ranging from the presence of multiple co-morbidities, increasing the complexity of management of the condition, and posing adherence challenges, through to the general lack of organized, systematic, mechanisms for monitoring and follow up for this patient population.

In contrast, Problem Solving Therapy (PST) is based on the observation that emotional symptoms are generally induced by problems of living, and has its theoretical roots in cognitive-behavioral approaches to mental disorders [34]. PST has been developed as a specific, collaborative treatment, with three main steps: first, patients' symptoms are linked with their problems; second, the problems are defined and clarified; and third, an attempt is made to solve the problems in a structured way with the aim of reasserting control over their lives, and it may be this regaining of control which lifts mood [35]. This process usually involves six sessions with a therapist, with a total contact time of less than four hours [36]. In randomized controlled trials, PST has been shown to be of comparable efficacy to antidepressant medication in the treatment of major depression when delivered by experienced general practitioners [37], and in the treatment of dysthymia (though not minor depression) in adults aged 18–59 when delivered by psychologists with PhDs [38].

The skills needed to deliver PST can be rapidly acquired by a range of health professionals including general practitioners, nurses and psychologists. In the USA it has demonstrable clinical effectiveness and cost-effectiveness when delivered by depression care managers as part of a package of care for older people [39,40]. Although it is not cost effective when delivered by specialist community psychiatric nurses for undifferentiated common mental health problems [41], PST has been shown to be effective when delivered in patients' own homes by trained facilitators with qualifications in psychology, nursing or allied health professions; and to be more effective than treatment as usual for people in five European countries with depressive and adjustment disorders identified through community survey [42].

### Interactional workability: the problem of congruence and disposal

The starting point for comparative analysis of the PST and Heart Failure Clinics trials is their *interactional workability*. This construct defines the dimensions of the immediate social context in which a complex intervention is enacted. In the UK, and elsewhere, there has been a shift to seeing the management of chronic illness as not only being more clinically effective and socially equitable when undertaken in primary care [43], but also as being more interactionally efficient because of its presumed continuity of care and holistic character [44]. This shift continues, although there has been a steady trend in UK primary care – and that of some other countries – towards more fragmented encounters focused through task, rather than person, oriented interactions and located in complex divisions of labor [45] and organizational settings [46].

In the case of PST, there appears to be considerable congruence between public preferences for psychological rather than drug treatments for depression [47] and those of its proponents. But in trials of PST, variable up-take of the treatment is a problem – 63% in the ODIN study [42]; 80% in the dysthymia study [38]; and 62% in the community psychiatric nurse study [41] – suggests that *in practice *congruence between professional and patient is far from complete, and that effective disposal is therefore unlikely in a substantial minority of cases. This suggests that not all patients share the expectations or meaning inherent in PST, or accept its legitimacy. This may, for example, be because some are more comfortable with treatment modalities such as medication that tend to absolve them from responsibility for their condition [48].

In considering its *interactional workability*, the question of whether PST confers an *interactional *advantage over existing therapies in accomplishing *congruence *in encounters between health professionals and patients, and hence more efficient *disposal *of work, is a complex one, because of the lack of observational data and the question of whether uptake of treatment is a good proxy measure. However, the hypothesis underlying PST, that emotional symptoms are generally induced by problems of living; its object, to increase patients' abilities to solve their problems; and provide a means to achieve this – six structured sessions with a therapist – completely rely on shared notions of legitimate conduct, and co-operation within clinical encounters. This means that *outcomes *of PST are a powerful proxy for its interactional workability.

We face a similar problem of proxy measures in the Heart Failure Clinics Trial [49]. This trial sought to respond to the problem of suboptimal care for older people with chronic heart failure. Other trials of specialist nurse interventions for heart failure patients have been undertaken and have demonstrated that such interventions can decrease hospital readmission rates, mortality and also improve quality of life [50]. The nurse-led heart failure clinics instituted in this trial involved the implementation of accepted guidelines in a structured way, a mode of practice with which practice nurses are accustomed and proficient. Patients too, are accustomed to nurse led primary care clinics where nurses substitute for family physicians [51-53]. It is also well established that patients respond well to practice nurses delivering routine chronic disease management services – nurses are viewed, for example, as being more holistic and informal in their approach, and as giving more time to interactional tasks [54].

Like PST, trial outcomes in nurse led heart failure clinics can be seen as a partial proxy for workability. Improved process measures, such as increased use of appropriate medications implies co-operation within clinical encounters. However, qualitative data draws attention to the lack of congruence between professionals and patients over the significance of CHF, and therefore over the importance of its clinical management [55,56]. Some patients simply did not understand the magnitude of their disease – either because it had not previously been disclosed to them in a way that they found comprehensible, or in a smaller number of cases, because they were unaware of their diagnosis, and thus failed to attend the clinic, meaning that congruence was not complete. Also, patients' lack of understanding of their condition meant that effective disposal *within *the clinic could be challenging as a good deal of remedial work was required on the part of the nurses delivering the service.

### Relational integration: accountability, confidence and trust

Health professionals' accounts of their practice typically take the important business of dealing with, and disposing of, patients' problems as a priority [57]. Equally, accounts of the design and outcomes of trials and other evaluations of complex interventions focus, for obvious reasons, on the intervention and its outcomes rather than on the processes by which these are obtained [5]. This makes it difficult to explore those aspects of complex intervention trials that involve 'hidden work' [58], and becomes important when we seek to understand their *relational integration*. Relational integration signifies the need to understand not only the possession and dispersal of knowledge needed to successfully utilize a complex intervention, but also the ways that those users understand and have confidence in the knowledge of people around them. In retrospective analyses like this one, the question of baseline knowledge and further training can be considered as a proxy measure for individual users – although it is of limited value in understanding processes of collective action.

When patients entered the heart failure clinics trial, the management protocols and techniques that nurses employed were based on clinical guidelines. But nurses required additional training and ongoing professional support to help them acquire and feel comfortable with their level of knowledge in this sphere. This improved their individual expertise and accountability by providing a strong *theoretical *background to heart failure – in other words the training was focused on the knowledge underpinning the intervention (the guideline) rather than its application in clinical interactions. This explains why nurses felt inadequately prepared for the practicalities of seeing patients with complex problems in the clinic. Thus, in the initial stages of the trial remedial work needed to be done, because professionals delivering the intervention expressed concerns about their confidence in delivering the intervention. The patients on the other hand, expressed confidence in the health professionals and felt that the intervention gave them greater confidence in managing their health. Qualitative data collected in this trial focused mainly on 'lay perceptions' of heart failure and lay evaluations of the clinic [56], but this data does indicate that professionals' accountability and confidence in knowledge seemed to be improved by the intervention.

Training needs cannot be a proxy for relational integration in the case of PST, since by definition entry into this work is dependent on specialized training and accreditation. As a treatment modality PST fits with, and even extends, established patterns of *knowledge *about the etiology and management of depression in primary care, and seems therefore to have greater potential for relational integration. In general, health professionals working in primary care tend to believe that problems of living are significant in the genesis of depression, and that knowledge of them is helpful in deciding on treatment options [41]. But *how *PST is integrated in any given intervention setting depends on who delivers the service. We know that there are differences in the way that knowledge is patterned within particular professional groups, and there are important debates to be had about what kinds of practitioner is best suited to deliver this kind of care. These debates become steadily more important as the epidemic of depression and anxiety seems to grow [59]. As it stands, questions about the focus of training and delivery of PST focus on: primary care physicians, who are limited in number, and have high patient credibility, but who work within significant constraints on time and effectiveness [60]; other healthcare professionals such as psychologists, nurses or counselors, whose interventions can be equally shown to be effective, but who are in equally short supply; or new breeds of professionals, such as depression care managers or graduate mental health workers [61].

### Skill-set workability: who should do this work?

Interactional workability and relational integration are constructs that refer to the *endogenous *factors affecting a complex intervention. This means that they are concerned with the immediate contexts in which different kinds of agents, and the objects of their agency, encounter each other. Thus, they are the immediate concern of those who propose and deliver complex intervention trials. But the wider context in which these encounters are set is no less crucial to their conduct. This involves *exogenous *factors which derive from the ecology in which they are set. These cannot be assumed, but merit an equal degree of attention from evaluators.

The first exogenous factor is reflected in the construct of *skill-set workability*, the extent to which a complex intervention is calibrated to an agreed set of skills in the health care division of labor. In the Heart Failure Clinics trial, this was relatively uncontentious. We have already noted that the delegation of medical work previously undertaken by primary care physicians to nurses is a normal feature of chronic disease management in the UK [53], and it figures prominently in questions of performance and quality [62,63]. This means that the division of labor within this service was congruent with established modes of working within primary care. Practice nurses delivered a guideline driven chronic disease clinic, and primary care physicians were drawn into this work when specific treatment changes were required or further advice needed to be sought. Despite this, some problems of communication between the practice nurses and general practitioners were noted. This may have been exacerbated by the fact that the training for the service was primarily, although not exclusively directed towards the nurses rather than the general practitioners. Thus there were occasions when nurses suggested treatment changes in accordance with the clinical guidelines, but changes were not accepted or enacted by the general practitioners. *Skill set workability *was therefore at times problematic from the perspective of at least one group of participants.

While the Heart Failure Clinics trial operated within the frame of a conventional model of nursing work, the delivery of PST is more contentious. It is by no means clear which type of health professional is most likely to deliver it, and thus how it might be calibrated to the professional division of labor in healthcare. Thus there remain unresolved questions about the allocation of work – and consequent levels of performance- between primary care physicians, other already existing healthcare professionals, and new members of primary healthcare teams. If general practitioners are to undertake this work for example, then the sense of professional autonomy will be high and surveillance is likely to be minimal; whereas if graduate mental health workers do so, autonomy is likely to be relatively low, and expectations for managerial surveillance consequently much greater. This means that the skill-set workability of PST is presently low in the settings suggested by the trials upon which this part of the paper focuses [37-40]. Although such resistance may well be related to concerns about job retention, it is more likely to be expressed in terms of *role uncertainty*. Existing healthcare professionals, when invited to comment on plans for a new collaborative care model for depression which would include case managers with skills in PST, express a wide variety of concerns. These include uncertainty about the professional values of the new workers, fears about their experience and competence, and statements about the need for education in non-specific skills necessary to develop a therapeutic alliance, as well as the knowledge and skills required for education, medication support and behavioral activation [64].

### Contextual integration: not just a problem of funding

It was noted above that conventional modes of reporting trials and locating qualitative studies within them provide limited information about processes. This becomes especially important when we focus our attention on the ways that a complex intervention 'fits' with the operational environment in which it is set. The Normalization Process Model proposes that a complex intervention will affect the mechanisms that link work to existing structures and procedures (its execution), and for allocating and organizing resources for them (its realization). But of course *trials *of complex interventions are normally carefully divided from the normal features of the environment in which they are set. One way to explain this is to say that they are, after all, only *temporary *and often unstable experiments that are overlaid on often very deeply embedded organizational and professional systems of practice. Furthermore, the demands of the trial protocol mean that this work of division is ongoing, so that the trial is not contaminated by those environmental features. But the integration of a complex intervention at an organizational level – its contextual integration – is much more than this. Other work [58], has shown this to be a crucial factor in the successful implementation of such interventions.

The absence of process evaluations that seek to understand the interactions between a complex intervention and its organizational context is important. In this area, proxies are very hard to come by. Perhaps the most obvious is the economic evaluation which contextualizes an intervention in terms of its costs, rather than its business processes. In our two case studies, we can only speculate about their potential for contextual integration. In the case of PST answers to this question will depend critically on what resource allocation models are employed. From the perspective of healthcare commissioners, it would appear most cost-effective to place the delivery of PST in the hands of new breeds of healthcare providers, such as depression care managers or graduate mental health workers, since these tend to be less qualified and hence less expensive. In a situation of expanding resource allocation, such a shift would be unlikely to be seen as prejudicial by general practitioners or existing healthcare professionals, but may rather be welcomed as an additional resource [65]. However in the more common situation where resource for healthcare is finite or even decreasing, any consequent shift in allocation would be likely to meet resistance from existing healthcare staff.

In the case of the Heart Failure Clinics trial the answer to this question may depend on the extent to which such systematic monitoring of heart failure patients in the community is seen as a central function of primary care both by the primary care professionals themselves and by external health care organizations. Importantly, this may be reflected in the degree to which such monitoring is rewarded through contractual arrangements in primary care. Thus the execution of such a service will depend to a large extent on how such work is valued or not at a health care systems level. This type of intervention is extremely labor intensive and the priority given to resourcing such initiatives are likely to be greatly influenced by prevailing economic factors.

## Conclusion

This paper has done three things. First, it has pointed to the need for sound process evaluations in trials of complex interventions and other implementation studies. Second, it has set out the basic features of a robust conceptual model that provides a sound explanatory basis for process evaluations. Third, it has applied the model retrospectively to trials of complex interventions in mental health and heart disease. We now turn to the question of *interaction *between complex interventions and their contexts. The most important consideration here is that the interventions discussed in this paper are competing with existing, deeply embedded, and thoroughly normalized modes of practice. While the trial assesses the relative effectiveness, and sometimes the relative cost-effectiveness of competing models of practice – the superiority of one over the other – process evaluations need to be concerned with their relative workability and integration.

The model invites evaluators to attend *equally *to four domains that have been shown to be of central importance to effective implementation. This is particularly important in considering how a complex intervention interacts with existing patterns of service organization, professional practice, and professional-patient interaction. Such an approach has a further important effect, which is to set out the implementation and integration of complex interventions as organizational or business processes that are *as important *to assess as clinical and cost effectiveness. It is important to note that this is not an appeal for more social science research within clinical and health technology assessment trials. Instead it suggests how trialists can engage with the complex dynamics of the settings in which they hope that their interventions will be implemented. This requires that the theory and methods of the social sciences are treated as integral elements in complex intervention trials. The justification for this may be found in the abundance of reports of clinically effectiveness interventions that have little hope of being implemented in real healthcare settings.

## Competing interests

The author(s) declare that they have no competing interests.

## Authors' contributions

CRM developed the theoretical model of normalization processes and with TLF modified it for this paper. FSM and CFD applied the model to specific trials. All authors contributed to writing the paper.

## Pre-publication history

The pre-publication history for this paper can be accessed here:


